# The Complex Role of the miR-17-92 Cluster in Stroke: Mechanistic Insights and Biomarker Potential

**DOI:** 10.3390/genes16060665

**Published:** 2025-05-29

**Authors:** Cornelia Braicu, Mihaela Molnar, Ekaterina Isachesku, Adrian Pană, Dafin Mureșanu, Stefan Strilciuc

**Affiliations:** 1 Department of Genomics, MEDFUTURE Institute for Biomedical Research, Iuliu Hațieganu University of Medicine and Pharmacy, 400337 Cluj-Napoca, Romania; cornelia.braicu@umfcluj.ro (C.B.); ekaterina.isachesku@umfcluj.ro (E.I.); strilciuc.stefan@umfcluj.ro (S.S.); 2 Department of Neuroscience, Iuliu Haţieganu University of Medicine and Pharmacy, 400012 Cluj-Napoca, Romania; mihaelamolnar1@gmail.com (M.M.); office@ssnn.ro (D.M.); 3 Center for Health Outcomes & Evaluation, Splaiul Unirii 45, 030126 Bucharest, Romania; 4 Research Unit, RoNeuro Institute for Neurological Research and Diagnostic, 400012 Cluj-Napoca, Romania

**Keywords:** ischemic stroke, miR-17-92 cluster, biomarkers

## Abstract

Stroke is a leading cause of morbidity and mortality worldwide, with ischemic stroke (IS) accounting for approximately 85% of cases. Recent research has highlighted the critical role of microRNAs (miRNAs), a class of small non-coding RNA molecules, in the pathogenesis of stroke. Among these, the miR-17-92 cluster and its paralogs have emerged as key regulators in the development of stroke pathology and the subsequent recovery processes. We emphasize their regulatory roles in key pathological processes, including inflammation, apoptosis, neuroprotection, and tissue repair. We provide an overview of these mechanisms to support the identification of novel miRNA-based therapeutic targets and to improve stroke diagnosis, treatment, and recovery strategies. Specific miRNAs, such as miR-19a, miR-18a, and miR-92a, contribute to processes including neurogenesis, axonal growth, and a reduction in neuronal apoptosis. The miR-17-92 cluster also offers potential therapeutic applications by targeting injury-induced pathways, such as modulating apoptosis, promoting axonal elongation, or inhibiting neurodegeneration. Preclinical studies have suggested their potential to enhance neural regeneration and promote functional recovery. Future research should further elucidate the regulatory mechanisms of the miR-17-92 members and their therapeutic potential to enhance stroke treatment strategies.

## 1. Introduction

A stroke is a disruption in blood supply to the brain, resulting in cell death in the affected cerebral territory. It is broadly classified into two major types: ischemic stroke (IS) and hemorrhagic stroke (HS). Ischemic stroke, accounting for approximately 85% of all cases, is caused by an obstruction, typically a thrombus or embolus, that blocks blood flow in an artery [1]. Conversely, hemorrhagic stroke occurs when a blood vessel ruptures, causing bleeding in or around the brain. Both types can lead to significant neurological damage and disability if not promptly treated, though they have distinct underlying mechanisms and treatment approaches [2,3].

Due to the myriad of known risk factors associated with stroke, including high blood pressure, diabetes mellitus, age, gender, and circulating cytokines that promote inflammation, the exact cause of the condition is still unknown [4]. The acute therapy for ischemic stroke (intravenous thrombolysis or mechanical thrombectomy) has a narrow therapeutic window, and the decision to administer treatment is based preponderantly on a clinical assessment [4,5].

A stroke induces widespread transcriptomic alterations, including the differential expression of genes, splicing events, and epigenetic modifications, which lead to changes in the coding and non-coding RNA profiles within the affected brain tissues [6]. These alterations affect critical cellular functions, including neuronal survival, synaptic plasticity, and inflammatory responses, thereby contributing to the complex pathophysiology of stroke. Coding RNAs, primarily messenger RNAs (mRNAs), undergo significant changes that affect protein synthesis, influencing processes such as neuronal repair and apoptosis. In recent years, there has been an increased interest in non-coding RNAs, including microRNAs (miRNAs) and long non-coding RNAs (lncRNAs), due to their critical regulatory roles in modulating gene expression, inflammatory signaling, and cellular stress responses [7].

miRNAs are small, non-coding RNA molecules, typically 21–25 nucleotides in length that regulate gene expression post-transcriptionally [8,9]. They function by binding to complementary sequences on mRNA, leading to mRNA degradation or the inhibition of translation [9,10,11]. miRNAs regulate multiple genes simultaneously, influencing key biological cellular processes, such as growth, differentiation, and death [1]. These non-coding transcripts modulate inflammation, neuroprotective, and tissue repair processes, influencing stroke outcomes and recovery [11,12,13,14]. Understanding these transcriptomic shifts is key to identifying novel biomarkers and therapeutic interventions. Analyzing miRNA expression patterns alongside transcriptomic changes can provide insights into the molecular mechanisms of stroke, help identify potential biomarkers for early diagnosis and prognosis, and uncover new therapeutic targets to mitigate stroke damage and enhance recovery [2,11,12].

An increasing number of studies are investigating the role of miRNAs in stroke pathogenesis, particularly their potential as diagnostic and prognostic biomarkers. This review focuses on the miR-17-92 cluster and its paralogs, synthesizing the current findings on their common and distinct functions in stroke. We emphasize their regulatory roles in key pathological processes, including inflammation, apoptosis, neuroprotection, and tissue repair. We provide an overview of these mechanisms to support the identification of novel miRNA-based therapeutic targets and to improve stroke diagnosis, treatment, and recovery strategies.

## 2. miR-17-92 Cluster and Its Paralogs

**Figure 1:**
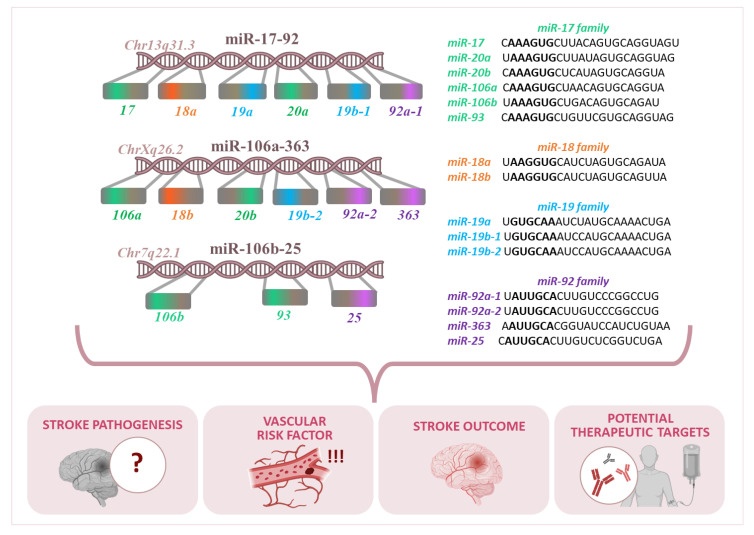
The role of the miR-17-92 cluster and its paralogs in different chromosomes and their relevance to stroke pathogenesis, vascular risk factors, and stroke outcomes. The three miRNA clusters shown are miR-17-92, miR-106a-363, and miR-106b-25.

The miR-17-92 cluster resides within an intron of the non-protein-coding gene MIR17HG, the host gene of the miR-17-92 cluster, also known as C13orf25 (Chromosome 13 open reading frame 25), on chromosome 13 (13q31.3) in the human genome. This specific cluster mainly contains four miRNA families: miR-17 (miR-17-5p, miR-20a, miR-20b, miR-106a, miR-106b, and miR-93), miR-18 (miR-18a and miR-18b), miR-19 (miR-19a, miR-19b-1, and miR-19b-2), and miR-92 (miR-92a-1, miR-92a-2, miR-25, and miR-363) [14,15,16,17], they are presented in Figure 1.

The human genome contains two paralogous clusters of the primary miR-17-92 cluster: the miR-106b/25 cluster and the miR-106a/363 cluster. The miR-106b/25 cluster, located on chromosome 7q22.1, resides within the 13th intron of the *MCM7* (Minichromosome Maintenance Complex Component 7) gene and includes three mature miRNAs: miR-106b, miR-93, and miR-25. The miR-106a/363 cluster, found on chromosome Xq26.2, comprises six mature miRNAs: miR-106a, miR-18b, miR-20b, miR-19b-2, miR-92a-2, and miR-363. While the miR-17-92 and miR-106b/25 clusters are highly expressed across various tissues, the miR-106a/363 cluster is generally expressed at lower levels (Table 1). Collectively, these three clusters encode up to 15-related miRNAs [14,18].

The miR-17-92 cluster and its paralogs are central to the critical pathways that govern cellular life and death decisions during normal development and with a malignancy [7,13,16]. A key focus is the continued elucidation of the whole network of targets regulated by these miRNAs. Adding to the complexity, the diverse functions of the miR-17-92 cluster and its paralogs in different physiological contexts likely involve different subsets of targets [18]. A broader issue is whether the functions of a given miRNA can be attributed to the strong regulation of a few dominant targets or the more subtle regulation of many targets simultaneously. miRNAs likely act through a variety of these mechanisms, some resulting from simple miRNA relationships and others from complex networks of gene expression changes [18]. Determining where the miR-17-92 cluster operates along this spectrum is a significant challenge in the research [2].

Various biological samples can be used to study the miR-17-92 cluster’s expression and its implications in stroke [13,16,17,19]. Blood samples, including plasma and serum, offer valuable insights into systemic changes and hold potential as biomarkers for diagnosis and prognosis. Cerebrospinal fluid (CSF), obtained via a lumbar puncture, provides information on local changes directly related to the central nervous system [20]. Additionally, in vitro cell cultures of neuronal or glial cells can be used to study the effects of miRNA in a controlled setting. Each sample type provides unique information and contributes to a comprehensive understanding of the roles of this transcript in stroke [20,21,22].

This unique capability allows this class of transcripts to exert control over multiple cellular pathways crucial for stroke. Each member of the miR-17-92 cluster and its paralogs might engage in nuanced interactions with distinct sets of target genes, influencing complex cellular networks essential for understanding and potentially treating stroke [17]. This versatility in target gene binding underscores the significance of these transcripts as key regulators for maintaining cellular homeostasis and responding to stroke challenges. Their role in these processes highlights their potential as valuable therapeutic targets for mitigating stroke impact and promoting recovery-related mechanisms [14,17,19,23]. Other studies have focused on the experimental modulation of specific miR-17-92 clusters and their paralogs altered by stroke to restore their normal expression levels or investigate their complex mechanistic roles through miRNA inhibition or replacement therapy [14,19,23].

## 3. Genetic Variations in the miR-17-92 Cluster and Its Paralogs: Implications for Stroke

Significant progress has been made in identifying the genetic markers of stroke risk, primarily by studying single-nucleotide polymorphisms (SNPs). SNPs within miRNAs, especially in their binding sites, can significantly influence miRNA-mRNA interactions. These genetic variations may alter an miRNA’s ability to bind to its target mRNA, disrupting miRNA-mediated gene repression. As a result, changes in gene expression due to miRNA-related single-nucleotide polymorphisms (miR-SNPs) can contribute to altered cellular functions related to stroke pathology [15,24,25]; this has been summarized in different bioinformatic databases, like MirSNPscore (https://www.bigr.medisin.ntnu.no/mirsnpscore/, accessed on 22 April 2025), PolymiRTS (http://compbio.uthsc.edu/miRSNP/, accessed on 26 April 2025), and MirSNP (http://bioinfo.bjmu.edu.cn/mirsnp/search/, accessed on 27 April 2025) [24].

miR-SNPs within the miR-17-92 cluster and its paralogs have been associated with stroke susceptibility and its outcomes, with the reported effects varying according to the studied population, likely due to genetic backgrounds and environmental factors [26]. Recent studies have identified several SNP loci in the precursors of miRNAs, including miR-146aC > G (rs2910164); miR-149T > C (rs2292832); miR-196a2T > C (rs11614913); and miR-499A > G (rs3746444), related to ischemic stroke, which was observed in a recent study of the Chinese population [27].

Polymorphisms in the miR-17-92 cluster, such as rs1491034, rs9301654, and rs982873, have been investigated for their potential association with clinical variables [7]. An Rs9301654 polymorphism in the promoter of the miR-17-92 cluster was found to be associated with susceptibility to IS [2]. Moreover, assessing the associations between these SNPs and serum lipid levels could provide insights into how genetic variations impact lipid-related pathways in stroke patients, potentially identifying new biomarkers for stroke risk; however, no direct correlation has been observed. In humans, germline loss or mutations of the miR-17-92 cluster have been found to lead to microcephaly and skeletal deformities [28,29].

These may exhibit population-specific frequencies and effects, influencing miRNA’s role in stroke susceptibility differently across different ethnic groups and environmental factors, as the data were related only to the Chinese population [2]. Therefore, understanding these population-specific variations in SNPs can provide valuable insights into the molecular mechanisms of stroke, help to identify more precise biomarkers, and guide the development of personalized prevention and treatment strategies tailored to different genetic backgrounds.

## 4. miR-17-92 Cluster: Implications of Altered Expression Levels as a Biomarker of Stroke

Changes in the expression of miRNAs in this cluster could serve as reliable biomarkers for stroke diagnosis and prognosis, offering insights into the extent of brain injury, recovery potential, and the likelihood of post-stroke complications [30]. Several notable findings underscore the role of the miR-17-92 cluster and its related paralogs as biomarkers for ischemic stroke are presented in Table 2.

Dong et al. evaluated the expression levels of the miR-17-92 cluster, revealing their downregulation in a serum study. In the same study, a four-miRNA diagnostic model was developed based on the representatives of this cluster (miR-18a-5p, miR-19a-3p, miR-19b-3p, and miR-20a-5p) [17]. A further analysis revealed an increased expression of these miRNAs in IS patients on day 7 compared to those measured on day 1. This suggests the dynamic regulation of these miRNAs throughout a stroke, reflecting ongoing pathophysiological changes, such as inflammation, tissue repair, or coagulation, during the recovery phase.

The PBMCs (peripheral blood mononuclear cells) of ischemic stroke survivors and a healthy control group were analyzed, and showed an increased expression of miR-17-5p and low levels of miR-19a-3p, suggesting a promising biomarker, and a correlation between an rs9301654 polymorphism and the expression of miR-19a-3p [2]. miR-17-5p was identified as an independent predictor, suggesting its potential utility in diagnostic assessments. Furthermore, the diagnostic value of miR-17-5p was notably enhanced when combined with miR-15a and miR-16 [31]. miR-17-5p was significantly higher in patients with acute cerebral infarction, indicating its potential role as a biomarker for early detection and diagnosis and recurrent disease [32].

Another study on leukocytes revealed a downregulation in the level of miR-19a and overexpression of miR-363 [33]. These miRNAs may play a significant role in regulating leukocyte gene expression during an acute ischemic injury. The immune response, leukocyte mobilization, and the development of thrombus are several processes involved in the pathological mechanism of stroke, besides the various gene implications, all of them being influenced by miR-19a and miR-363 expression, increasing the interest in using them as biomarkers and potential therapeutic targets [33].

miR-93 levels were significantly reduced in the plasma and neutrophils of those with IS [34]. Neutrophil miR-93 levels were positively correlated with the Barthel Index 7 days after a stroke, serving as an indicator for diagnosing and predicting functional recovery in acute stroke patients. Additionally, miR-93 levels were negatively correlated with the expression of TNF-α and IL-10 [34].

A profiling study focused on the identification of a specific miRNA pattern in acute ischemic stroke (AIS) versus transient ischemic attack patients (TIA) revealed 11 differentially regulated miRNAs, among them two representants of the miR-17-92 cluster (miR-20a-5p and miR-18a-5p) [35].

A study conducted on 238 patients with acute ischemic stroke highlighted the importance of miR-92a expression; the patients with white matter hyperintensities and increased levels of miR-92a had an increased risk of developing post-stroke depression [36].

**Table 2:** Some relevant examples related to the miR-17-92 cluster and its related paralogs as biomarkers for IS.

Type of Stroke	Expression Level	Biological Specimens and Approach Used for Evaluation	Relevant Findings of the Study	Reference
IS and HS	↓ miR-17-5p, ↓ miR-18a-5p, ↓ miR-19a-3p, ↓ miR-19b-3p ↓ miR-20a-5p, ↓ miR-92a-3p	Serum from IS patients (n = 58) and healthy controls (n = 50); GSE117064 cohort (173 IS cases and 1612 HC cases); days 1 and 7.	Diagnostic value for IS; moderate discrimination ability for distinguishing IS from HC.	[17]
IS	↓ miR-17-5p, ↓ miR-17-3p, ↓ miR-18a-3p, ↓ miR-18a-5p, ↓ miR-19b-3p, ↓ miR-19a-3p, ↓ miR-92a-3p	GSE110993, from IS (n = 20) and control group (n = 20).	circ_0011474-hsa-miR-20a-5p/hsa-miR-17-5p-CDKN1A ceRNA regulatory axis in IS.	[2]
IS	↑ miR-17-5p, ↓ miR-19a-3p	PBMC; IS (n = 398) and control group (n = 397).	Diagnostic role.	[7]
IS	↑ miR-17-5p	Serum; IS (n = 106) and healthy control group (n = 102).	MiR-17-5p an independent predictor; a higher diagnostic value of miR-17-5p in combination with miR-15a and miR-16.	[31]
IS	↑ miR-17-5p	Plasma from IS patients (n = 83) and healthy controls (n = 37).	Diagnostic and recurrence role.	[32]
IS	↓ miR-19a	Leukocytes; IS (n = 24) and control group (n = 24).	miR-19a is part of an altered signature, along with downregulated miR-122, miR-148a, let-7i, miR-19a, miR-320d, and miR-4429 and overexpressed miR-363 and miR-487b; involved in thrombus formation.	[33]
IS and TIA	↓ miR-18a-5p, ↓ miR-20a-5p	Serum; IS (191) and TIA (61).	Part of an 11-miRNA signature to discriminate IS versus TIA.	[35]
IS	↓ miR-93	Plasma and neutrophils; IS (n = 33) and control group (n = 20).	Diagnostic and prognostic role; correlated with neurological function score; correlated with the expression of TNFα and IL10.	[34]
IS	↓ miR-19a, ↑ miR-363	Leukocytes; IS (n = 24) and vascular risk factor controls (n = 24).	miR-363 part of altered signature along with overexpression of miR-122, miR-148a, let-7i, miR-19a, miR-320d, and miR-4429 and downregulation of miR-487b.	[33]
IS	↓ miR-92a	Serum; IS (70) and control group (n = 25).	miR-92a part of altered signature along with downregulation of miR-375 and overexpression of miR-134.	[37]

PBMC: peripheral blood mononuclear cell; IS: ischemic stroke; HS: hemorrhagic stroke; TIA: transient ischemic attack patients; AIS: acute ischemic stroke; TNFα: tumor necrosis factor-α; IL10: interleukin-10; ceRNA: competing endogenous RNA.

## 5. miR-17-92 Cluster and Its Paralogs Affect Key Physiological and Pathological Processes

Stroke involves critical physiological and pathological processes, including ischemia, cellular injury, oxidative stress, and inflammation [38,39]. Alterations of the transcriptomic profile can modulate cell death and survival, influence inflammation, impact the blood–brain barrier integrity, and affect neuronal growth and synaptic plasticity in acute stroke damage, impacting treatment outcomes and recovery [38].

### 5.1. miR-17-92 Cluster and Its Paralogs Affect Cell Death/Survival and Differentiation

Cell death, survival, and stem cell renewal are influenced by the miR-17-92 cluster [13,29,40]. miR-20 is involved explicitly in proper neural development. It was found that miR-20a/b influences the developmental-stage-specific mean and variation in cyclin D1 protein levels in a feedback regulatory network. This control underpins the failsafe process that enables cortical progenitors to decide whether to proliferate or differentiate. The downregulation of this transcript raises their fluctuating spectrum of expression and the intermediate expression levels of cyclin D1, disrupting the equilibrium between progenitor proliferation and differentiation. While the miR-20a/b–cyclin D1 network’s parts could influence themselves, the exact regulation of cyclin D1 expression is more complicated and warrants additional investigation [41].

The overexpression of certain representants of this cluster has been shown to provide neuroprotection by inhibiting apoptosis and promoting cell survival pathways [13,28,29,40]. The inhibition of miR-19a offers neuroprotection against ischemic stroke by modulating glucose metabolism and reducing neuronal apoptosis. This process involves enhancing the cellular energy supply and stabilizing metabolic pathways, crucial for neuronal survival during ischemic events. By mitigating apoptosis, miR-19a inhibition helps preserve neuronal integrity and function, ultimately improving outcomes and reducing the damage associated with ischemic stroke [33].

PTEN, a phosphatase and tensin homolog deleted on chromosome 10, constitutes one of the identified genes targeted by the miR17-92 cluster [42], and it has been found to have detrimental effects on embryonic neural stem cell growth and viability [28]. Furthermore, the high expression of the cluster inhibits PTEN proteins and reduces elevated mammalian target of rapamycin (mTOR) [29,42] in cortical neurons, therefore enhancing axonal growth [28]. Removing the transcribed PTEN protein gene promotes axon regrowth in adult corticospinal neurons following spinal cord damage [29].

### 5.2. miR-17-92 Cluster and Its Paralogs’ Regulation of Angiogenesis

The growth of new blood vessels has been demonstrated to stimulate the growth of new nerve endings. Therefore, it is essential to protect blood vessels as much as possible to encourage angiogenesis, mainly for improving recovery after a central nervous system injury [40]. The miR-17-92 cluster in endothelial cells (ECs) regulates fetal and adult angiogenesis through gene expression modulation. Extensive studies have conclusively demonstrated that vascular endothelial growth factor-A (VEGF) induces the expression of the miR-17-92 cluster, showing that VEGF has a significant role in stimulating angiogenesis, especially in cases of central nervous system (CNS) injury [40]. The miR-17-92 cluster has been implicated in regulating angiogenesis and forming new blood vessels. This is crucial for recovery after a stroke, as new vessel formation can help restore blood supply to ischemic areas [40].

### 5.3. miR-17-92 Cluster and Its Paralogs Are Involved in the Blood–Brain Barrier (BBB)

Changes in the expression of the miRNAs in this cluster can impact the integrity of the BBB, influencing its permeability and potentially contributing to cerebral edema [43,44].

Numerous biological processes are regulated by miR-17-5p in endothelial cells [44,45,46,47]. miR-17-5p plays a role in bacterial meningitis by disrupting the BBB [48]. Agomir-17-5p reduced BBB permeability, while antagomir-17-5p increased it. TARBP2, an RNA-binding protein (RBP), was bound to SNHG7, extending its half-life. The reduced expression of miR-17-5p negatively regulated NFATC3 post-transcriptionally, leading to increased NFATC3 levels. Additionally, SNHG7 acted as a molecular sponge for miR-17-5p, further regulating NFATC3 expression. NFATC3 suppressed the expression of tight junction (TJ) proteins by functioning as a transcription factor. This TARBP2/SNHG7/miR-17-5p/NFATC3 pathway suggests a potential mechanism underlying BBB alterations in Alzheimer’s disease [48].

### 5.4. miR-17-92 Cluster and Its Paralogs’ Regulation of Thrombus Formation

miRNAs regulate thrombus formation, influencing coagulation and platelet function [33]. miR-19a is particularly interesting as it targets the tissue factor pathway inhibitor (TFPI), reducing the tissue factor activity essential for initiating the coagulation cascade. By modulating the TFPI, miR-19a may help prevent excessive clot formation [33]. Additionally, miR-19a also regulates SERPINE1, the gene encoding plasminogen activator inhibitor-1 (PAI-1), which inhibits tissue plasminogen activator (tPA) and urokinase (uPA). This suggests that miR-19a could influence fibrinolysis by controlling the activity of endogenous tPA in ischemic stroke. Moreover, miR-19a targets other key factors in the coagulation pathway, such as coagulation factor 3 (F3), further underscoring its potential role in thrombus regulation during ischemic events [33].

### 5.5. miR-17-92 Cluster and Its Paralogs Affect Neuroinflammation, Neurogenesis, and Neural Repair

The miR-17-92 cluster modulates neuroinflammation by targeting the genes involved in inflammation, potentially affecting the severity of the inflammatory response and secondary injury after a stroke [49]. Key findings on the role of miR-17-92 cluster members in experimental stroke therapy are presented in Table 3. The altered expression of miRNAs in this cluster can influence cytokine production, thereby impacting the inflammatory environment in the brain and potentially influencing recovery outcomes [7].

The miR-17-92 cluster plays a role in neuronal growth and differentiation. Alterations in its expression can affect neurogenesis, which is essential for brain repair and functional recovery after a stroke. The miRNAs in this cluster can influence synaptic plasticity, vital for cognitive and functional recovery following stroke [49].

Neurogenesis is closely linked to angiogenesis in the germinal zones of the adult brain, where neural progenitor cells (NPCs) are located near endothelial cells, and both processes begin synchronously. In vitro, endothelial cells influence neuronal differentiation by secreting soluble factors [29]. The close association of brain blood vessels with the basal lamina of the subventricular zone (SVZ), where neurogenesis starts, provides anatomical evidence for the interaction between vasculogenesis and neurogenesis in the adult brain. These findings have led to the recognition of a neurovascular niche, highlighting the mutual support between neurons and vascular endothelial cells [40]. In NPCs of the ischemic subventricular zone, members of the miR-17-92 cluster have been found to modulate their proliferation and survival after stroke [40].

The overexpression of the miR-17-92 cluster in cortical neurons significantly boosted axonal outgrowth, while inhibiting miR-19a in distal axons suppressed it. This effect was linked to reduced PTEN and increased phosphorylated mTOR in axons. Conversely, attenuating miR-19a increased PTEN and inactivated mTOR. The overexpression of PTEN reversed the outgrowth promoted by miR-19a, and blocking mTOR signaling with LY294002 or rapamycin abolished the effect. These findings suggest that miR-19a modulates axonal outgrowth by locally regulating PTEN and mTOR in axons [29].

The increased expression of miR-106a/b stimulates the generation of neurons from neural stem cells (NSCs) by shifting the differentiation lineage; this is regulated viaTp53inp1-Tp53-Cdkn1a [50].

The neuroprotective role of miR-20a-3p in stroke was shown in a rat model; a decreased expression of miR-20 was associated with a poor outcome after stroke and was found in the astrocytes of middle-aged females and adult and middle-aged males, who typically have this outcome [41].

The neuro-functional recovery observed after stroke was enhanced by miR-17-92-cluster-enriched MSC exosomes, which may be linked to increased axonal extension and myelination [43], and mediated through the activation of the PTEN-mediated PI3K/Akt/mTOR pathway [43] or via PI3K/AKT/VEGFA [45].

A major contributor to cardiovascular pathology, such as atherosclerosis, restenosis, and hypertension, are vascular smooth muscle cells (VSMCs). Compared with other cells, they may transition from contractile (differentiation) to proliferative (de-differentiation) states as a reaction to various pathogenic stimuli. Kee et al. used a rodent carotid injury to demonstrate the upregulation of miR-18-5p in the incipient stage of endothelium damage. VSMC differentiation was promoted through the suppression of syndecan4 by miR-18a-5p [51]. As a member of the heparan sulfate proteoglycan, syndecan4 has substantial implications for cardiovascular pathology, including hypertrophy of myocardial cells, acute coronary syndrome, and arterial restenosis following angioplasty [51]. Vascular homeostasis is believed to be regulated by miR-92a, mainly because of an increased expression in endothelial cells [52]. Inhibiting endothelial miR-92a can reduce neointimal lesion development, increase reendothelization, and enhance functional recovery after vascular damage in a mouse [52].

**Table 3:** Some relevant examples of miR-17-92 cluster members in stroke therapy in in vitro models.

MiRNA Species	Experimental Model	Observation	Reference
miR-17-5p	arterial endothelial cells	Promotes endothelialization and facilitates the vascular repair of aneurysms via PTEN-mediated PI3K/AKT/VEGFA pathway.	[44]
miR-19a	primary murine cortical neurons	Axonal outgrows via PTEN and mTOR axis.	[29]
miR-20a/b	spinal cord injury (SCI); astrocytes and microglia culture	Cell proliferation, apoptosis, and neuronal differentiation; miR-20a/b regulates the developmental stage of cortical neurons by targeting cyclin D1 and HspB1; enhances neurite outgrowth in cortical neurons and axonal growth and neuronal branching in hippocampal neurons. Provides neuroprotection and ameliorates IS.	[41]
miR-25	human SH-SY5Y and IMR-32 cells; OGDR model	Downregulated in an OGDR model related to the released Fas/FasL; regulated cell death pathways.	[53]
miR-93	OGD of BV2 microglial cells; miR-93 mimic	Promotes cell proliferation in OGD and induces G1 phase cell-cycle arrest.	[34]
miR-106b	neural stem/progenitor cells	Regulates proliferation and differentiation via the Tp53inp1-Tp53-Cdkn1a axis.	[49]

OGD: oxygen–glucose deprivation (OGD); OGDR: oxygen–glucose deprivation/reperfusion.

## 6. miR-17-92 as a Therapeutic Strategy for Stroke

The miR-17-92 cluster plays an important regulatory role in key signaling pathways in ischemic stroke pathophysiology. Table 4 outlines the observed effects of the miR-17-92 cluster members in various in vivo models of IS and TBI, including MCAO, MACAO, and TBI models. It highlights changes in expression levels and biological outcomes, such as improved functional recovery, reduced neuronal loss, enhanced axonal remodeling, and modulation of signaling pathways like PTEN/PI3K/Akt/mTOR. The findings demonstrate the therapeutic potential of this miRNA cluster for promoting neuroprotection and regeneration.

Targeting the miR-17-92 cluster using gene therapy approaches, such as antagomirs (anti-miRNA oligonucleotides) or miRNA mimics, could provide new therapeutic strategies to modulate the expression of these miRNAs and potentially reduce stroke-induced damage. The ongoing research aims to translate these findings into clinical applications, offering hope for improved stroke outcomes.

miR-25 overexpression downregulated the Fas protein in an OGDR (oxygen–glucose deprivation/reperfusion) model, and an siRNA-mediated Fas knockdown similarly inhibited apoptosis. Conversely, Fas overexpression negated miR-25’s protective effects. These findings suggest that miR-25 inhibits cerebral ischemia/reperfusion (I/R) injury-induced apoptosis by downregulating the Fas/FasL pathway, offering a potential therapeutic target as an apoptotic regulator [53].

In an experimental study by Zhang et al. on cultured cortical neurons harvested from embryonic rats using a microfluidic chamber, the overexpression of miR-19 was shown to enhance axonal outgrowth compared with the culture with an empty vector [29]. A recent study revealed that the overexpression of the miR-17-92 cluster in cultured ischemic neural progenitor cells or in the SVZ of ischemic animals significantly increased cell proliferation. After the procedure, neurons were cultured in a microfluidic chamber for 5 days, and miR-18a and miR-19a were assessed. The low expression of miR-19a, but not miR-18a, inhibited axonal outgrowth, indicating its important role in axonal elongation [29]. Also, it is believed that after a stroke, there is an upregulation of miR-18a and miR-19a, enhancing the cell proliferation and differentiation of neuronal progenitor cells into neuroblasts [54]. The downregulation of miR-19a is correlated with an increase in apoptotic cells. By inhibiting individual members, miR-18a and miR-19a reduced proliferation and increased cell death [54], playing an essential role in mediating neural progenitor cell function via the Shh signaling pathway [54].

In a zebrafish model, the importance of miR-18a during cerebral and retinal embryonic development was assessed. It was observed that miR-18a is a key factor in regulating NeuroD and photoreceptor differentiation. In fish missing miR-18a, photoreceptors developed quicker without abnormalities in the retinal structure, indicating that miR-18a suppression may be a therapeutic target. Also, it was shown that the timing of the spatiotemporal pattern of photoreceptor differentiation modulated by miR-18a is needed for normal retinal development [55].

The administration of exosomes engineered to enrich the miR-17-92 cluster markedly reduces neuroinflammation and neuronal cell loss, enhances neurogenesis and angiogenesis, and significantly improves functional recovery in traumatic brain injury (TBI) compared to control exosomes. By tailoring the contents of exosomes to boost neural plasticity and recovery, MSC-derived exosomes enriched with specific miRNAs could provide an effective and potentially superior alternative to stem cell therapy for neural injuries or diseases. miR-17-92-enriched exosomes promoted sensorimotor functional recovery in rats after a traumatic brain injury [56].

The inhibition of miR-19a protects neurons from ischemic stroke by modulating glucose metabolism and reducing neuronal apoptosis. By targeting Adipor2, miR-19a inhibits glucose uptake and promotes neuronal apoptosis. By regulating glucose metabolism and preventing cell death, miR-19a inhibition helps maintain neuronal function and resilience during ischemic events [57]. ADIPOR2 is the functional effector of miR-19a-3p during a cerebral ischemic injury [57].

**Table 4:** Summary of the functional roles of the miR-17-92 cluster members in IS and TBI using in vivo models.

Pathology	Cluster Member	In Vitro System	Observation	Reference
IS	↑ miR17-92 cluster	Animal model	↓ PTEN and mTOR regulate axonal growth.	[28]
IS	↑ miR-17-92 cluster	Enriched exosomes; MACO models	Increase functional recovery after stroke via PTEN-mediated PI3K/Akt/mTOR; regulate axon remodeling.	[43]
IS	↑ miR-17-92	TBI model/miR-19-92 exosomal delivery	miR-17-92-enriched exosomes reduce hippocampal neuronal cell loss in rats after TBI and enhance sensorimotor function.	[56]
IS	miR-17-92	MACAO	miR-17-92-enriched exosomes increase neural plasticity in the IBZ and promote neurite outgrowth and myelination vis the PTEN/PI3K/Akt/mTOR pathway.	[43]
TBI	↑ miR17-92 cluster	Traumatic brain injury (TBI) model; engineered exosomes carrying the elevated miR-17-92 cluster	Improve sensorimotor functional recovery; improve spatial learning and memory; reduce hippocampal neuronal cell loss; reduce brain inflammation; ↓ CD68+ microglia/macrophages and GFAP+ astrocytes in the LBZ and DG (anti-neuroinflammation).	[56]
IS	↑ miR-19a ↑ miR-18a	SVZ neural progenitor cells; MACAO model	Overexpression of miR-17-92 cluster in MACAO versus non-MACAO model; mediates neural progenitor cell function and Shh signaling.	[54]
IS	↓ miR-19a-3p	I/R and OGD model; primary neurons and astrocytes	↓ miR-19a-3p in rat neurons; ↑ miR-19a-3p in R/OGD models; MiR-19a inhibits glucose uptake and promotes neuronal apoptosis by targeting *Adipor2*.	[57]
IS	↓ miR-20a	MCAO; TBI injury	Downregulated in the first 24 h of cerebral ischemia in the blood and brain; intravenous injection of miR-20a-3p after stroke within the first 4h improved patient outcomes after MCAO compared to the control group.	[41]
IS	↓ miR-20a	OGD/R and MACAO	miR-20b regulates neuron apoptosis and ischemic brain injury by targeting TXNIP.	[58]

TBI: traumatic brain injury; I/R: in vivo ischemic/reperfusion neuronal injury; OGD: oxygen–glucose deprivation (OGD); PTEN: phosphatase and tensin homolog deleted on chromosome 10; PI3K: phosphoinositide 3-kinase; mTOR: mammalian target of rapamycin; Fas/FasL pathway; MCAO: model of middle cerebral artery occlusion; SVZ: subventricular zone; ADIPOR2: adiponectin receptor 2; TXNIP: thioredoxin-interacting protein; GFAP: glial fibrillary acidic protein; LBZ: lesion boundary zone; IBZ: ischemic boundary zone; DG: dentate gyrus; MSCs: mesenchymal stem cells.

## 7. Conclusions

**Figure 2:**
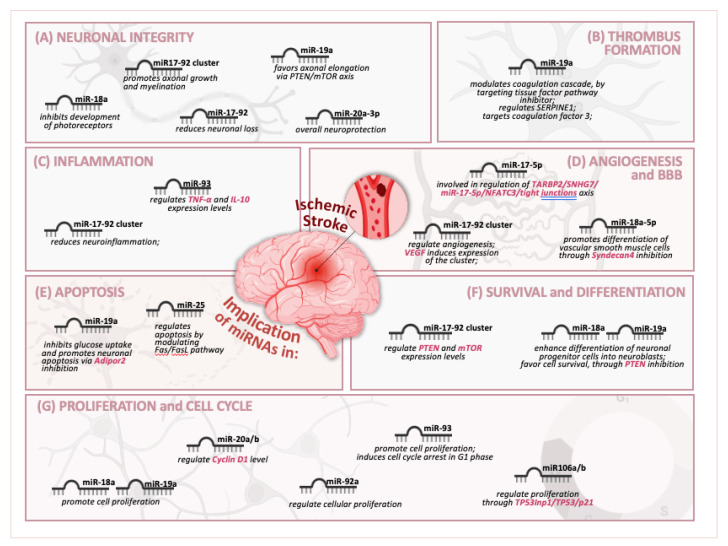
Complex regulatory roles of miR-17-92 cluster in key biological processes associated with ischemic stroke recovery and pathology. (**A**) Neuronal Integrity: miRNAs play an important role in maintaining neuronal structure and function after ischemic stroke. For example, miR-18a, miR-19a, and miR-20a-3p are implicated in axonal elongation, development of photoreceptors, and overall neuronal protection, contributing to neuronal regulation in context of ischemic injury. (**B**) Angiogenesis and Blood–Brain Barrier (BBB): Several miRNAs have been found to regulate vascular functions and integrity. miR-18a-5p promotes differentiation of vascular smooth muscle cells by inhibiting Syndecan4, further supporting vascular remodeling after stroke. Other miRs from miR17-92 cluster promote angiogenesis or regulate atherosclerosis. (**C**) Inflammation: Inflammation is a key component of stroke pathology, and several miRNAs modulate inflammatory responses. Studies have shown that miR17-92 cluster is implicated in reducing neuroinflammation and controlling pro- and anti-inflammatory signals. (**D**) Metabolism: miRNAs help modulate metabolic processes, especially glucose metabolism, critical during stroke recovery. For example, miR-19a influences glucose metabolism, while miR-20a/b and miR-23 may regulate glycogenic shifts related to astrocytes. (**E**) Apoptosis: Regulation of programmed cell death is vital in limiting damage after ischemia. miR-19a promotes neuronal apoptosis by inhibiting Adipor2, playing a role in determining fate of damaged neurons. (**F**) Survival and Differentiation: miRNAs influence both survival and differentiation of neural cells. miR17-92 cluster regulates key survival pathways, possibly by modulating PTEN and mTOR expression. For example, miR-18a and miR-19a enhance differentiation of neuronal progenitor cells into neuroblasts and promote cell survival by inhibiting PTEN. (**G**) Proliferation and Cell Cycle: miRNAs also control cell proliferation and cell cycle. Specific miRNAs (e.g., miR-18a, miR-19a, miR-20a/b, and miR-23) modulate process of cellular proliferation through regulation of cell cycle progression factors, such as cyclins, cyclin-dependent kinases inhibitors, or tumor suppressors. Overall, figure underscores multifaceted roles of miR-17-92 cluster in regulating various cellular and molecular processes involved in brain’s response to ischemic stroke, offering insight into potential therapeutic targets for neuroprotection and recovery.

The miR-17-92 cluster demonstrates significant potential applications as a biomarker for stroke, with promising benefits for the early detection, prognosis, disease monitoring, and understanding of stroke pathophysiology, as observed for Figure 2. However, future research must focus on validating these miRNAs in clinical settings and incorporating them into current diagnostic and therapeutic strategies to enhance stroke patient management.

The members of the miR-17-92 cluster are of great importance in stroke. Although some of their functions and mechanisms are fully understood, the latest research on miRNAs is important for understanding disease development and identifying potential therapeutic targets. As the biological functions and molecular mechanisms of the miR-17-92 cluster are gradually uncovered, our findings may help identify novel biomarkers and molecular targets for treating patients.

## Figures and Tables

**Table 1:** Genomic localization of the human miR-17-92 and its paralogs, miR-106a-363, and miR-106b-25 clusters; data for MiRNAs were downloaded from https://www.mirbase.org/, accessed on 22 April 2025.

Genome Context	Name	Accession	Chromosome	Start	End	Strand	Confidence
chr13: 91350605–91350688	hsa-mir-17	MI0000071	chr13	91350605	91350688	+	High
	hsa-mir-18a	MI0000072	chr13	91350751	91350821	+	High
	hsa-mir-19a	MI0000073	chr13	91350891	91350972	+	High
	hsa-mir-19b-1	MI0000074	chr13	91351192	91351278	+	High
	hsa-mir-20a	MI0000076	chr13	91351065	91351135	+	High
	hsa-mir-92a-1	MI0000093	chr13	91351314	91351391	+	High
chrX: 134170198–134170278	hsa-mir-18b	MI0001518	chrX	134170041	134170111	−	High
	hsa-mir-19b-2	MI0000075	chrX	134169671	134169766	−	High
	hsa-mir-20b	MI0001519	chrX	134169809	134169877	−	High
	hsa-mir-92a-2	MI0000094	chrX	134169538	134169612	−	High
	hsa-mir-106a	MI0000113	chrX	134170198	134170278	−	High
	hsa-mir-363	MI0000764	chrX	134169378	134169452	−	High
chr7: 100093560–100093643	hsa-mir-25	MI0000082	chr7	100093560	100093643	−	High
	hsa-mir-93	MI0000095	chr7	100093768	100093847	−	High
	hsa-mir-106b	MI0000734	chr7	100093993	100094074	−	High

## Data Availability

No new data were created or analyzed in this study. Data sharing is not applicable to this article.

## Abbreviations

The following abbreviations are used in this manuscript:

TBI	traumatic brain injury
IS	ischemic stroke
HS	hemorrhagic stroke
I/R	in vivo ischemic/reperfusion neuronal injury
OGD	oxygen–glucose deprivation (OGD)
OGDR	oxygen–glucose deprivation/reperfusion
PTEN	phosphatase and tensin homolog deleted on chromosome 10
PI3K	phosphoinositide 3-kinase
mTOR	mammalian target of rapamycin
miRNAs	microRNAs
MCAO	model of middle cerebral artery occlusion
SVZ	subventricular zone
ADIPOR2	adiponectin receptor 2
TXNIP	thioredoxin-interacting protein
GFAP	glial fibrillary acidic protein
LBZ	lesion boundary zone
IBZ	ischemic boundary zone
DG	dentate gyrus
MSCs	mesenchymal stem cells
